# Supercritical CO_2_ Extraction of Phytocompounds from Olive Pomace Subjected to Different Drying Methods

**DOI:** 10.3390/molecules26030598

**Published:** 2021-01-23

**Authors:** Graziana Difonzo, Antonella Aresta, Pietro Cotugno, Roberta Ragni, Giacomo Squeo, Carmine Summo, Federica Massari, Antonella Pasqualone, Michele Faccia, Carlo Zambonin, Francesco Caponio

**Affiliations:** 1 Department of Soil, Plant and Food Sciences, Food Science and Technology Unit, University of Bari Aldo Moro, Via Amendola, 165/A, I-70126 Bari, Italy; giacomo.squeo@uniba.it (G.S.); carmine.summo@uniba.it (C.S.); antonella.pasqualone@uniba.it (A.P.); michele.faccia@uniba.it (M.F.); francesco.caponio@uniba.it (F.C.); 2 Department of Chemistry, University of Bari Aldo Moro, Via Amendola, 165/A, I-70126 Bari, Italy; antonellamaria.aresta@uniba.it (A.A.); roberta.ragni@uniba.it (R.R.); carlo.zambonin@uniba.it (C.Z.); 3 Department of Biology, University of Bari Aldo Moro, Via Amendola, 165/A, I-70126 Bari, Italy; pietro.cotugno@uniba.it (P.C.); federica.massari@uniba.it (F.M.)

**Keywords:** olive pomace, upcycling, hot-air drying, freeze drying, supercritical fluid extraction, by-product, tocopherols, phytosterols, antioxidants

## Abstract

Olive pomace is a semisolid by-product of olive oil production and represents a valuable source of functional phytocompounds. The valorization of agro-food chain by-products represents a key factor in reducing production costs, providing benefits related to their reuse. On this ground, we herein investigate extraction methods with supercritical carbon dioxide (SC-CO_2_) of functional phytocompounds from olive pomace samples subjected to two different drying methods, i.e., freeze drying and hot-air drying. Olive pomace was produced using the two most common industrial olive oil production processes, one based on the two-phase (2P) decanter and one based on the three-phase (3P) decanter. Our results show that freeze drying more efficiently preserves phytocompounds such as α-tocopherol, carotenoids, chlorophylls, and polyphenols, whereas hot-air drying does not compromise the β-sitosterol content and the extraction of squalene is not dependent on the drying method used. Moreover, higher amounts of α-tocopherol and polyphenols were extracted from 2P olive pomace, while β-sitosterol, chlorophylls, and carotenoids were more concentrated in 3P olive pomace. Finally, tocopherol and pigment/polyphenol fractions exerted antioxidant activity in vitro and in accelerated oxidative conditions. These results highlight the potential of olive pomace to be upcycled by extracting from it, with green methods, functional phytocompounds for reuse in food and pharmaceutical industries.

## 1. Introduction

The growing interest in the use of natural functional molecules for consumer purposes in view of human health and environmental protection has favored a series of research studies on the extraction of various phytocompounds from natural raw plants. The management of wastes and by-products of large-scale agricultural processes also represents a key factor in reducing production costs, providing benefits related to their reuse [1,2]. Huge amounts of residues resulting from food industries (i.e., olive oil, wine, and dairy product chains), in some cases, induce issues of pollution related to their phytotoxicity and high costs for their removal [3,4,5].

Olive pomace is a semisolid by-product of olive oil production, whose amount and physicochemical properties depend on the adopted oil extraction method. The extraction process of virgin olive oils involves (**i**) olive crushing, (**ii**) olive paste malaxation, and (iii) olive oil separation from the other phases (solid and water phases) by centrifugation. Various generations of decanters have been designed and set up over time, among which the main ones used are the two-phase (2P) and three-phase (3P) water saving decanters; to a lesser extent percolation and pressure systems—now in disuse for economic and oil quality reasons—are adopted for oil separation. [6]. Olive pomace includes a variable small portion of residual oil that can be recovered by solvent extraction after drying the olive pomace [7]. This recovery process generates another waste, known as dry olive cake, suitable as a fuel [3,7]. Due to the presence of residual oil components, olive pomace is a useful source of a variety of phytocompounds, such as polyphenols, fatty acids, natural pigments (such as chlorophylls and carotenoids), tocopherols, phytosterols, squalene, and volatile and aromatic compounds [8,9]. The extent of lost bioactive compounds during olive oil processing and their existence in waste streams have important implications for the valorization of olive pomace, which can represent a valuable source rather than a waste. The distribution in olive and waste streams (olive mill wastewater and pomace) of predominant lipophilic bioactive molecules, such as squalene, β-sitosterol, and α-tocopherol, has been reported in the literature [10], as well as the loss of lipophilic phytocompounds during the industrial virgin olive oil production [11]. 

Among residual oil biocomponents, phytosterols comprise molecules with structures and functions similar to cholesterol. They have long been recognized as molecules capable of having beneficial effects on human health, such as reduction of serum cholesterol levels and prevention of cardiovascular and tumoral diseases [12,13,14,15]. Hence, phytosterols have generated great interest among pharmaceutical and cosmetics industries, promoting industrial production of steroid drugs and cosmetic creams or lipsticks from plant waste materials, such as cellulose and vegetable oils [2,16]. Durante et al. [17] used the response surface methodology to optimize the extraction of bioactive molecules from patè olive cake and showed health-related potential of the obtained extracts. Furthermore, the effect of hydrothermal pretreatments, including the use of steam and subcritical water, for the recovery of sterols, phenols, and oil was evaluated [18]. 

Squalene is another compound present in the waste from oil processing. It is a natural isoprenoid acting as an intermediate metabolite in the biosynthesis of cholesterol and vitamin D. It does not suffer from lipid peroxidation and is responsible, as an antioxidant, for protecting DNA against oxidative damage in mammalian epithelial cells [2,19].

Tocopherols and tocotrienols (vitamin E) are lipophilic phenols existing in α-, β-, γ-, and δ-forms in different percentages in vegetable oils and their processing scraps. The most abundant natural vitamin E isoform is an α-tocopherol representing up to 90% of total form, even if different factors, such as the geographic location of olive trees, may influence its concentration [20,21]. It is also the most active form of vitamin E in mammals [22]. Tocopherol was extracted from olive husk using supercritical carbon dioxide (SC-CO_2_), and the optimal extraction conditions were determined by Gracia et al. [23]. 

The need for more ecosustainable and viable processes has led food industries and scientists to develop alternative processes in line with the adoption of green and eco-friendly extraction methods [24]. Such processes are based on the use of renewable plant sources and green solvents, as well as on the reduction of energy consumption and working steps, to valorize agroindustrial by-products [25,26]. On this ground, supercritical fluid extraction (SFE) used as a sample preparation and purification step for analytical purposes provides several advantages compared to conventional extraction methods since it uses supercritical fluids (SFCs), such as carbon dioxide, methanol, ethane, n-pentane, ethene, and n-butene. Their physicochemical properties can be modified by controlling pressure and temperature, with or without a cosolvent, usually ethanol [27,28], to recover bioactive molecules from different matrices [8]. Since carbon dioxide is not dangerous for human health, is generally recognized as safe (GRAS) by FDA (Food and Drug Administration) and EFSA (European Food Safety Authority), and is environment friendly, it is the most employed supercritical fluid, even in large-scale systems, to obtain a pure product, without traces of solvent and in a small amount of time. Various studies have been reported on supercritical fluid extraction with or without a cosolvent of phytosterols, essential oils, pigments [29], and mainly polyphenols from blueberry, cranberry, and raspberry wastes [30]; wine industry by-products [31]; pistachio hull [32]; olive leaves [33]; and orange, apple, and tomato pomace [34,35,36,37]. The SFE of polyphenols has been further carried out from olive pomace [38].

Drying methods induce a significant effect on plant matrices, affecting several features of phytocompounds [39]. Freeze drying is generally preferred since it avoids heat damage and reduces losses of volatile components and textural changes, whereas the high temperature and long time required with hot-air drying adversely affect the texture, color, and flavor of bioactive compounds [39,40]. However, freeze drying is an expensive process not completely in line with the aims of economic and environmental sustainability [41].

In this work, we focused on extracting with supercritical carbon dioxide (SC-CO_2_) functional phytocompounds from olive pomace samples obtained from the two most common industrial olive oil production processes based on the separation step with two- and three-phase decanters. In particular, we evaluated the effects of freeze and hot-air drying, investigating the best drying conditions for upcycling olive pomace samples to carry out on them ecofriendly SC-CO_2_ extraction of functional phytocompounds. 

## 2. Results and Discussion

### 2.1. Extraction of Lipophilic Phytocompounds

Olive pomace samples obtained from the two most common industrial olive oil production processes, based on two and three-phase decanters, namely 2P and 3P samples, were subjected to freeze and hot-air drying, leading to F2P (freeze-dried olive pomace from the two-phase extraction system), F3P (freeze-dried olive pomace from the three-phase extraction system), H2P (hot-air-dried olive pomace from the two-phase extraction system), and H3P (hot-air-dried olive pomace from the three-phase extraction system). SC-CO_2_ extraction was then carried out according to a method reported in the literature for recovery of lipophilic compounds from residual olive husk by supercritical fluids [23,42]. Three different fractions were produced to extract tocopherols, squalene, and phytosterols as reported in the Section 3, and their amounts are reported in Table 1.

As expected, the main compound in the tocopherol fraction is α-tocopherol, whose identification and quantification were carried out by ultra-high performance liquid chromatography-fluorescence detector (UHPLC-FLD) by comparing the relative external standard and calibration curves. The F2P sample had the highest content of α-tocopherol and specifically for the extract obtained from 2P olive pomace, the drying method significantly affects the content of extracted α-tocopherol (*p* < 0.001), this being 39% less in H2P. Conversely, no significant difference in the content of α-tocopherol was found in the F3P and H3P samples, demonstrating that the dehydration method has no effect in this case. This result could be related to the different moisture contents of the 2P and 3P pomace samples. In fact, the relative humidity content is 65% in 2P and 51% in 3P. So 2P requires more time for hot-drying at 120 °C than 3P (35 vs. 23 min). In virgin olive oil, the amount of α-tocopherol, which represents 90% of the total tocopherols, is in the 55–370 mg kg^−1^ range and it was estimated that more than 50% is recovered in oil and only 10% is lost in olive pomace [11]. A similar range was obtained in SFE extracts reported in our work, with the lowest concentration of α-tocopherol in 3P samples (45 and 63 mg·kg^−1^ in H3P and F3P, respectively). As previously reported, no difference in the presence of α-tocopherol in oils was observed with respect to the type of decanter used, but the content is highly variety dependent [43,44].

Squalene is a natural triterpene and an important intermediate of sterol and hopanoid biosynthesis in various types of cells, from bacteria to human. The richest source of squalene, however, is shark liver oil, which has been traditionally used as a source of this lipid. It is widely present in nature, and substantial amounts are found in palm oil, wheat-germ oil, amaranth oil, rice bran oil, and olive oil [45]. In our olive pomace extracts, the highest content of squalene was found in H2P samples (5712 mg·100 g^−1^), while no statistical difference was found in the other extracts. Dehydration by hot-air drying positively affects the squalene content in 2P olive pomace, whereas no difference was found between hot-air drying and freeze drying for 3P samples, as also was the result of the two-way ANOVA, showing no significant effect of D*T interaction on the results. The amount of extracted squalene was the highest compared to the amounts of the other compounds (up to about 6000 mg·100 g^−1^). The highest extraction efficiency was observed for squalene compared to α-tocopherol and β-sitosterol from olive and pomace samples. Squalene is mainly concentrated in the pulp and is present in free form in the cell membrane, whereas α-tocopherol and β-sitosterol are concentrated in the seed of the olive fruit (inside the stone wall) and are bound to the cell membrane [11]. 

β -sitosterol is one of the several phytosterols with a chemical structure similar to that of cholesterol; is generally considered as a safe, natural, and effective nutritional supplement; and has been shown to have many potential benefits, such as antioxidant, antimicrobial, angiogenic, antioxidant, immunomodulatory, and antidiabetic effects. [46]. In the phytosterol-enriched fraction, β-sitosterol was detected by comparison with the retention time of the relative standard and also literature data [18], as reported in the Section 3.

As shown in Table 1, a higher content of β-sitosterol in the 3P versus 2P sample was found (195 vs. 120 mg·100 g^−1^ as mean values, respectively), and the quantity was not affected by the drying method both in 2P and 3P samples. In fact, literature reports that phytosterols are crystalline solids at room temperature [47] and are considered stable compounds, which undergo only limited degradation during heating [11]. 

### 2.2. Pigments Fraction and Antioxidant Evaluation

**Figure 1:**
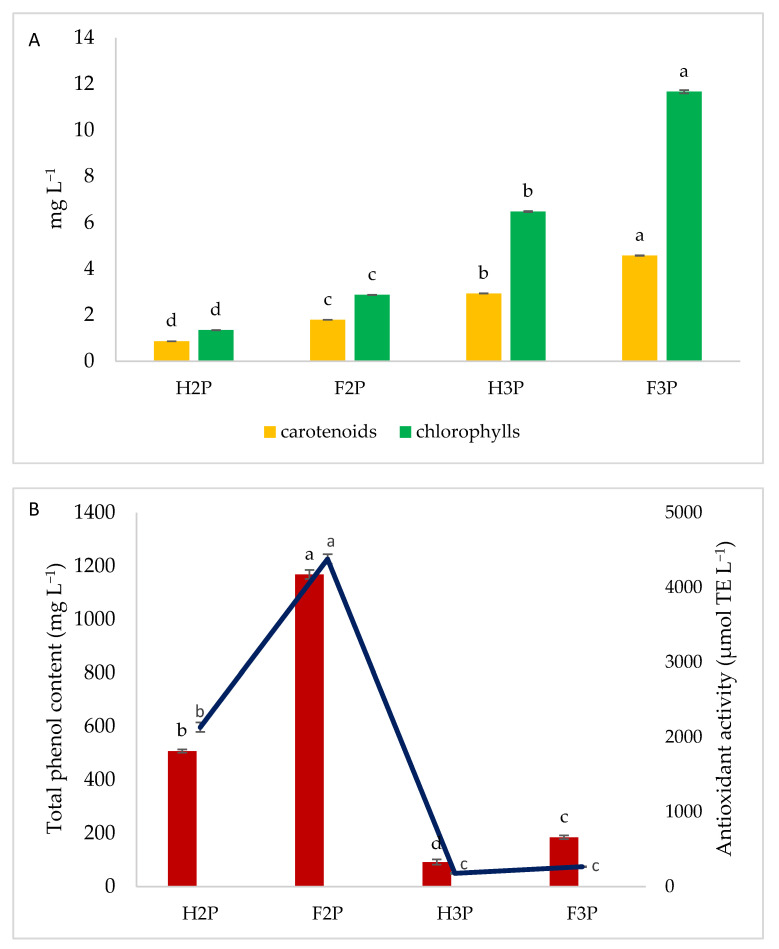
(**A**) Carotenoid and chlorophyll spectrophotometric determination. (**B**) Total phenol content (bars) and antioxidant activity evaluation (line). a,b,c,d-Different letters indicate significant statistical differences at *p* < 0.05. Abbreviations: H2P, hot-air-dried olive pomace from the two-phase extraction system; F2P, freeze-dried olive pomace from the two-phase extraction system; H3P, hot-air-dried olive pomace from the three-phase extraction system; F3P, freeze-dried olive pomace from the three-phase extraction system.

An additional fraction enriched with carotenoid and chlorophyll pigments was obtained. The trend was the same for both compounds, showing the highest values in F3P samples (4.57 vs. 11.65 mg·L^−1^ for carotenoids and chlorophylls, respectively). As for 2P and 3P samples, hot-air drying negatively affects the content of these compounds (Figure 1A). Literature works have reported that after oven drying, some plant compounds, such as carotenoids and chlorophylls, decrease between 4% and 100% [48]. Moreover, heat treatment can lead to the formation of pheophytin from chlorophylls because magnesium in chlorophyll can be easily displaced by two hydrogen atoms [49]. Some authors have reported that the drying process induces loss in the β-carotene content, which can be attributed to the drying temperature [50], and during thermal processing, an increase in β-carotene *cis*-isomer formation with increasing treatment intensity was observed [51].

Carotenoids are among the most common natural pigments, and several different compounds have been characterized up to now, with β-carotene playing a major role in the protection of plants against photooxidative processes [52]. For this reason, the antioxidant activity was assayed in the pigment-enriched fraction by means of the DPPH (2,2-diphenyl-1-picrylhydrazyl) test (Figure 1B).

Interestingly, an opposite trend was observed with respect to carotenoid quantification, showing the best results for F2P and H2P samples. Moreover, as for 2P and 3P olive pomace, significantly higher results of antioxidant activity were detected in freeze-dried samples. Since the SFE method was cosolvent (ethanol) assisted, the presence of polyphenols in this fraction was verified by the Folin–Ciocalteu assay. As shown in Figure 1B, the results were in accordance with those of antioxidant activity, thus leading to the conclusion that presumably polyphenols contribute more than carotenoids to the antioxidant power. Moreover, results confirm that the higher content of polyphenols in 2P than in 3P olive pomace can be due to the presence of a major content of olive mill wastewater in 2P olive pomace compared to 3P [6].

**Figure 2:**
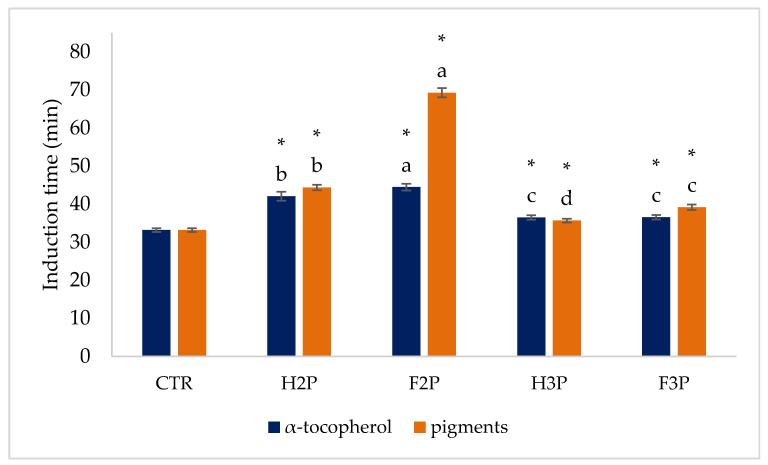
Oxidative stability test on purified oils with added α-tocopherol and pigment fractions. a,b,c-Different letters indicate significant statistical differences at *p* < 0.05. *-Asterisks indicate the significant difference on comparing samples with control by Dunnett’s test. Abbreviations: CTR, control; H2P, hot-air-dried olive pomace from the two-phase extraction system; F2P, freeze-dried olive pomace from the two-phase extraction system; H3P, hot-air-dried olive pomace from the three-phase extraction system; F3P, freeze-dried olive pomace from the three-phase extraction system.

Figure 2 shows the results of the oxidative stability test performed on purified olive oil with added α-tocopherol and pigment-/polyphenol-enriched fractions. In fact, these phytocompounds are known to be good antioxidants, with high potential to be used as additives in foods [53]. Moreover, there is a significant commercial interest in the development of functional foods containing a range of bioactive components and offering multiple health benefits [54]. α-Tocopherol, the most abundant and biologically active form of vitamin E, is a lipophilic antioxidant and can reduce the risk of many chronic diseases associated with oxidative stress, including cancer, cardiovascular disease, and neurological and endocrinological disorders, showing thus a good potential in the biomedical field [55].

All samples show a significant increase in the induction time with respect to the control and thus higher stability against forced oxidation. Data detected for α-tocopherol are in accordance with those of quantification (Table 1), whereas for the pigment-enriched fraction, the results are in line with the trend obtained for total phenol content determination. Thus, also in the condition of oxidative stability test, polyphenols are more effective than pigments in exerting an antioxidant power.

### 2.3. Multivariate Analysis

**Figure 3:**
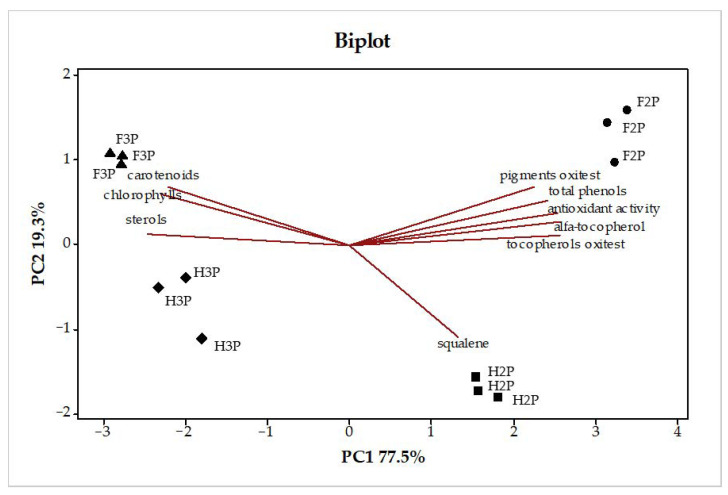
Biplot resulting from the principal component analysis performed on the whole dataset. Abbreviations: H2P, hot-air-dried olive pomace from the two-phase extraction system; F2P, freeze-dried olive pomace from the two-phase extraction system; H3P, hot-air-dried olive pomace from the three-phase extraction system; F3P, freeze-dried olive pomace from the three-phase extraction system.

Figure 3 shows the biplot of the principal component analysis (PCA) obtained from the whole data set. The first principal component (PC), which explains more than 77% of the total variability, wraps up almost all the information of the dataset, corresponding roughly to that of 6–7 variables out of 9. The second PC has around 19% of the residual variability. The combination of data sets results in a clear-cut differentiation of 2P from 3P olive pomace samples on PC1. According to the results discussed previously, the marker parameters that contribute to the differentiation of 2P and 3P samples are α-tocopherol, total phenol content, antioxidant activity evaluation, pigments, and sterols. These variables showing higher absolute loadings on PC1 are those having the greatest influence on sample differentiation. The parameters with positive loadings on PC1 are related with 2P olive pomace samples and those with negative loadings with 3P samples. PC2 explains the separation between freeze drying and hot-air drying methods, for which all the parameters appear to be related with freeze-dried samples; only squalene shows a different behavior, being mostly related with H2P samples. 

## 3. Materials and Methods

### 3.1. Sampling 

Olive pomace was obtained by two olive oil extraction systems: the two-phase (2P) decanter (Mori-Tem, Oliomio Sintesi, Florence, Italy) and the three-phase (3P) decanter (Pieralisi Vanguard 4704, Jesi, Italy). Olives from the Apulia region (Italy) were harvested during the crop season 2019–2020 and processed. Moreover, two different drying methods were adopted: freeze drying, with equipment (De Mori, Milano, Italy) conditioned at −60 °C at 0.8 mbar pressure, and hot-air drying in a TCF 120 ventilated oven (Argo-Lab, Carpi, Italy) at 120 °C to achieve a final moisture between 3 and 5%. Thus, the samples were classified as (i) freeze-dried olive pomace from the two-phase extraction system (F2P), (ii) freeze-dried olive pomace from the three-phase extraction system (F3P), (iii) hot-air-dried olive pomace from the two-phase extraction system (H2P), and (iv) hot-air-dried olive pomace from the three-phase extraction system (H3P). 

### 3.2. Chemicals

Carbon dioxide (SCF grade) from Rivoira (Milan, Italy) was used for supercritical fluid extraction. LC-grade ethanol from Sigma Aldrich (Milan, Italy) was used as a CO_2_ modifier. All chemicals were purchased from Merck (Darmstadt, Germany) and solvents from Carlo Erba (Milan, Italy).

### 3.3. Supercritical Fluid Extraction

#### 3.3.1. Apparatus 

The extraction apparatus is a Spe-ed SFE (Applied Separations, Allentown, USA) system supplied from operating with carbon dioxide as a supercritical fluid (SC-CO_2_). It consists of a pump for CO_2_, an oven, a collection module, and a control module. The oven module consists of a controlled-temperature extraction chamber, containing one or two metal vessels to perform two extractions simultaneously due to the double fluid line. A thermo-couple sensor allows temperature detection on the vessel surface. The pump module for CO_2_ is driven by air (7 bar) in continuous mode generated by a low-noise compressor. The supply module also consists of a cooling and conditioning system for the pump head (chiller and external recirculating bath). The collection module, equipped with a heated micrometric valve, allows SC-CO_2_ extracts to be collected. The control module allows you to set up and control all parameters. Moreover, the system is equipped with a 40P pump (Knauer Wissenschafìliche Geràte GmbH Berlin, Germany), a pump head for a maximum flow rate of 10.00 mL/min, and a maximum pressure of 700 bar, for a cosolvent-assisted extraction; in particular, the cosolvent line includes a pressurization branch and a T valve to introduce the modifier into the line of the SC-CO_2_. Supercritical fluid extraction is performed by means of repetitive static and dynamic steps under an optimized set of parameters.

#### 3.3.2. Experimental Design for Supercritical Fluid Extraction

SC-CO_2_-based extraction of functional lipophilic compounds was performed on freeze-dried and hot-air-dried olive pomace samples, both obtained with two-phase and three-phase extraction procedures. In particular, a different set of parameters was adopted for the extraction, according to the developed experimental plan, as reported in Table 2. To obtain extracts rich in tocopherols, the extraction was performed at 40 °C, CO_2_ pressure of 250 bar, and a flow rate of 1 liter per minute (LPM), operating three cycles, each cycle of 3 min for both static and dynamic steps. The extraction was performed at 60 °C, 350 bar, and 1 LPM, by performing three cycles, i.e., 3 min of static and 10 min of dynamic cycle, to recover high amounts of carotenoids and chlorophylls. In this case, a cosolvent, such as ethanol, was also required during the dynamic phase at a flow rate of 0.1 mL·min^−1^. To obtain extracts rich in sterols, extraction at 60 °C, 250 bar, and 1 LPM was performed three times, with a static time of 3 min and a dynamic time of 15 min. A cosolvent flow of 0.4 mL·min^−1^ was used during the dynamic step. To recover a high amount of squalene, extraction was carried out by using a temperature of 40 °C and pressure and flow rate for carbon dioxide equal to 90 bar and 1 LPM, respectively, for three cycles, composed of static and dynamic periods, each one of 3 min.

For SC-CO_2_ extraction, 10 grams of olive pomace samples were loaded into a metal cylinder vessel. The sample was premixed with a proper amount of Ottawa sand as the dispersant material. Each extraction was performed in triplicate.

### 3.4. Chemical Characterization of Extracts

#### 3.4.1. Tocopherols

Tocopherols were determined by reverse phase-ultra high performance liquid chromatography-fluorescence detector (RP-UHPLC-FLD) [56]. The samples were dissolved in 1 mL of 2-propanol and filtered. Then, 20 µL were injected into the UHPLC system (Dionex Ultimate 3000 RSLC, Waltham, MA, USA) equipped with an HPG-3200 RS pump, a WPS-3000 autosampler, a TCC-3000 column compartment, and a FLD-3400 RS fluorescence detector and using a Dionex Acclaim 120 C18 analytical column (150 × 3 mm i.d.) with a particle size of 3 μm (Thermo Scientific, Waltham, MA, USA). The mobile phase consists of a mixture of acetonitrile and methanol (1:1 *v*/*v*) at a constant flow rate of 1 mL·min^−1^ in isocratic elution mode. The FLD detector was set at an excitation wavelength of 295 nm and at an emission of 325 nm. The quantification was reached by means of the external calibration obtained with standard solutions of α-tocopherol in the concentration range of 10–1000 mg·kg^−1^.

#### 3.4.2. β-Sitosterol and Squalene

The gas chromatography-flame ionization detector (GC-FID) method was performed, as reported by Secmeler et al. [18], with some modifications. A mixture of the silylating reagent hexamethyldisilazane (HMDS), the catalyst trimethylchlorosilane (TMCS), and the polar solvent pyridine in 2:1:10 volumetric ratio (*v*/*v*/*v*) was added to the extracts and then they were blended. α-Cholestanol was added to the mixture as the internal standard. After that, the derivatization reaction was completed at 60 °C for 60 min the mixture was centrifuged at 12,000 rpm for 4 min to precipitate and discard ammonium chloride salt, a by-product of silylation reaction, which causes decomposition and interfering peaks [57]. The clear supernatant phase was transferred to a GC vial and injected into a GC-FID. GC analysis was carried out using a GC System Agilent Technologies 7890 (Agilent Technologies Inc., Santa Clara, CA, USA) equipped with an FID and an HP-5 capillary column (30 m × 0.32 mm id, 0.25 μm film thickness, Agilent Technologies Inc., Santa Clara, CA, USA). Flow rates of FID gases (air and hydrogen) and the make-up (helium) gas were 136 mL·min^−1^, 35 mL·min^−1^, and 45 mL·min^−1^, respectively. Helium was used as a carrier gas (1.0 mL·min^−1^) with a split injection (60:1). The analyses were carried out in programmed temperature mode holding the initial temperature of 270 °C for 23 min and raising it to 290 °C with a ramp rate of 4 °C·min^−1^ followed by a 12 min hold at 290 °C. The identification of the compounds was made by comparison with literature data [18] and with the retention time of the relative standards. Quantitative analysis was performed using the internal standard method.

#### 3.4.3. Chlorophylls and Carotenoids

The total chlorophyll content was determined spectrophotometrically using the method reported by Paradiso et al. [58]. Extracts were filtered (nylon, 0.45 µm) and transferred into spectrophotometric cuvettes. The absorbance was recorded at 661.6 nm and 644.8 nm with a Cary 60 UV-VIS system (Agilent Technologies, Santa Clara, PA, USA) and used to calculate the concentration of each type of chlorophyll, using the following formulas:

chl_a_ (mg·L^−1^) = 11.24 A_661.6_ − 2.04 A_644.8_ (1)

chl_b_ (mg·L^−1^) = 20.13 A_644.8_ − 4.19 A_661.8_ (2)

where A_n_ is the absorbance of the extract at a wavelength of *n* nm. The total chlorophyll was calculated as the sum of chlorophyll a (chl_a_) and chlorophyll b (chl_b_). Carotenoids were spectrophotometrically measured at 449 nm [56], and the results were expressed as mg·L^−1^ of extract of β-carotene.

#### 3.4.4. Total Phenol Content and Antioxidant Activity Evaluation

The determination of the total phenol content (TPC) was performed by the Folin–Ciocalteu method according to Tarantino et al. [59] with some modifications. An amount of 20 µL of the pigment fraction (chlorophylls and carotenoids) was added to 980 µL of ddH_2_O and 100 µL of the Folin–Ciocalteu reagent. After 3 min, 5% Na_2_CO_3_ solution was added, following which the mixture was incubated at room temperature for 60 min. The absorbance was read at 750 nm using a Cary 60 spectrophotometer (Agilent, Cernusco, Italy). The TPC was expressed as gallic acid equivalents (GAE) in mg·L^−1^ of extract. 

The DPPH (2,2-diphenyl-1-picrylhydrazyl) assay was performed, according to Ranieri et al. [60], on the same fraction by preparing a solution of DPPH 0.08 mM in ethanol, and the results were expressed in µmol Trolox equivalents (TE) L^−1^ of extract.

An accelerated oxidation test (oxitest) was performed by means of RapidOxy (Anton Paar, Blankenfelde-Mahlow, Germany), a microprocessor-controlled automatic testing device for quick measurements of the oxidative stability of lipid matrices, in response to forced oxidation with an increase in temperature and O_2_ pressure. The induction time of the sample is measured as the time needed for a 10% drop in the oxygen pressure. The set parameters were the following: *T* = 140 °C, *P* = 700 kPa. Purified olive oil, obtained as reported by Difonzo et al. [61], was used as the lipid matrix. The pigment- and tocopherol-enriched fractions were added to purified olive oil at concentrations of 100 mg·g^−1^ and 100 µg·g^−1^, respectively. Each sample was analyzed in triplicate.

### 3.5. Statistical Analysis

The results were expressed as the mean ± the standard deviation (SD) of three different trials. Significant differences between the values of all parameters were determined at *p* < 0.05, according to the two-way analysis of variance (ANOVA) followed by the Tukey test for multiple comparisons. Dunnett’s test and principal component analysis (PCA) were performed for the multiple comparison with control and for the multivariate approach on the whole dataset, respectively. All these statistical analyses were performed by the Minitab Statistical Software (Minitab Inc., State College, PA, USA).

## 4. Conclusions

In our study, two different types of olive pomace were subjected to supercritical fluid extraction, being considered a promising source of interesting compounds, such as tocopherols, β-sitosterol, squalene, chlorophylls, and carotenoids. Moreover, the effects of two different drying processes on the extraction efficiency of the selected phytocompounds were evaluated. In most cases, freeze drying preserved the functional molecules (especially α-tocopherol, carotenoids, chlorophylls, and polyphenols) better, whereas no difference between freeze and hot-air drying was detected for squalene. Hot-air drying did not compromise the content of β-sitosterol. Higher amounts of α-tocopherol and polyphenols were extracted from 2P olive pomace, while β-sitosterol, chlorophylls, and carotenoids were more concentrated in 3P olive pomace. Finally, tocopherols and pigment/polyphenol fractions exerted antioxidant activity in vitro, as in accelerated oxidative conditions. Overall, our results highlight the potential of the by-product olive pomace to be upcycled by extracting from it, with green methods, functional phytocompounds for reuse in food and pharmaceutical fields. 

## Figures and Tables

**Table 1:** Quantitation of the main lipophilic compounds in different samples of olive pomace.

	*p*-Value			Samples			
Compounds	D	T	D*T	H2P	F2P	H3P	F3P
α-Tocopherol (mg·kg^−1^)	0.000	0.000	0.000	259.9 ± 16.7 ^b^	424.5 ± 4.2 ^a^	44.6 ± 7.4 ^c^	63.4 ± 4.9 ^c^
Squalene (mg·100 g^−1^)	0.000	0.001	0.161	5712 ± 126 ^a^	4201 ± 375 ^b^	4316 ± 603 ^b^	3450 ± 49 ^b^
β-Sitosterol (mg·100 g^−1^)	0.929	0.000	0.451	123.9 ± 23.1 ^b^	114.8 ± 14.4 ^b^	191.1 ± 21.7 ^a^	198.9 ± 9.9 ^a^

^a,b,c^ Different letters mean a significant difference at *p* < 0.05 (two-way ANOVA and multiple comparisons by the Tukey test); *D* (drying) and *T* (typology) were considered as variables. Abbreviations: H2P, hot-air-dried olive pomace from the two-phase extraction system; F2P, freeze-dried olive pomace from the two-phase extraction system; H3P, hot-air-dried olive pomace from the three-phase extraction system; F3P, freeze-dried olive pomace from the three-phase extraction system.

**Table 2:** Parameters for SC-CO_2_ extraction of phytocompounds from olive pomace.

Phytocompounds	Temperature	Pressure	Flow Rate	Cycle and Time	Cosolvent
Tocopherols	40 °C	250 bar	1 LPM	3 cycles: 3 min for both static and dynamic steps	No cosolvent used
Carotenoids and chlorophylls	60 °C	350 bar	1 LPM	3 cycles: 3 min for static and 10 min for dynamic	Ethanol at 0.1 mL·min^−1^
Sterols	60 °C	250 bar	1 LPM	3 cycles: 3 min for static and 15 min for dynamic	Ethanol at 0.4 mL·min^−1^
Squalene	40 °C	90 bar	1 LPM	3 cycles: 3 min for both static and dynamic steps	No cosolvent used
